# Burnout hazard in teachers results of a clinical-psychological intervention study

**DOI:** 10.1186/1745-6673-6-37

**Published:** 2011-12-22

**Authors:** Ralf Wegner, Peter Berger, Bernd Poschadel, Ulf Manuwald, Xaver Baur

**Affiliations:** 1 Institute for Occupational and Maritime Medicine, Seewartenstrasse 10, 20459 Hamburg, Germany; 2 Hardtwaldklinik II, Bad Zwesten, Germany; 3 Chair of Occupational Health, University Medical Center Hamburg-Eppendorf, Germany

## Abstract

**Background:**

The study investigates whether established in-patient therapy for teachers with burnout results in long-acting success and whether gender gaps and differences between teachers of different school levels exist. According to our knowledge, our study is the most extensive inpatient intervention study on the burnout of a defined occupational group, i.e., teachers.

**Methods:**

200 teachers participated, 150 took part in a later performed katamnestic survey.

The Maslach Burnout Inventory (MBI) was used and work-related data were recorded. The days of incapacity for work and the percentage of teachers endangered by burnout decreased, which supports the long-term success of the treatment.

**Results:**

Significant differences between males and females and between teacher levels were found. However, the differences between teacher levels only showed up before treatment. Because males only underwent treatment at a more severe stage, further efforts in persuading males to start therapy earlier are needed.

**Conclusions:**

The proven and long-term success of the performed intervention could have greater effects if people, especially males, undergo treatment more frequently. Our results are based on selectively high proposition of teachers of advanced age. Thus it is possible that the long term effect of the intervention, particularly on retirement age, is greater when the intervention is started earlier. Regular burnout tests could help to identify risk cases among teachers at an early stage and to offer a therapeutic intervention.

## Introduction

More than three decades ago, Freudenberger [1] described the exhaustion syndrome of socially engaged employees for the first time and termed it burnout. Since then, numerous publications on this topic from various occupational groups have become available [2-6]. However, few studies on the treatment of burnout in socially engaged employees exist. Various recommendations [7-10] and reports on short-term positive results of in-patient burnout therapies [11] are available, but long-term effects have scarcely been documented by extensive studies.

The most frequent reasons for early retirement among teachers are psychical diseases, with account for 70% of early retirements. Therefore timely intervention is essential to reduce these disorders and consequent early retirement in this group. The objective of this study was to investigate whether an established psychotherapeutically oriented stationary treatment supplemented by a job-specific intervention shows long-acting success and whether gender gaps and differences between high school teachers and teachers of other levels exist. Gender and school type variations in the extent of burnout have been described in previous studies [12-14].

The criteria of this evaluation were the number of employees who resumed teaching, the reduction of periods of incapacity for work and the improvement of emotional exhaustion according to the subscale of the Maslach Burnout Inventory [15,16].

## Methods

The group to be tested consisted of 200 teachers (civil servants) from Germany (aged between 27 and 64 years; mean + standard deviation (SD) 51.1 + 6.7 years) who voluntarily underwent a stationary treatment for emotional exhaustion in a psychotherapeutically oriented clinic between 2001 and 2007. The patients were referred to the clinic by external medical specialists who considered an ambulant therapy insufficient. Patients with an acute psychosis or a florid addiction were excluded from the investigation. Of the admitted teachers (134 females and 66 males), 100% participated in this study. Thirty-four were high-school teachers (18 men, 16 women), while 166 worked at other schools (48 men, 118 women).

At the beginning of the stationary treatment, a clinical diagnosis according to ICD 10 (International Classification of Diseases, 10^th ^revision) was performed and a questionnaire was completed. A follow-up mail survey was conducted one year after treatment termination at the earliest (mean ± SD 2.0 ± .7 years). The same questions answered in the first examination were answered in the follow-up.

The location of the clinic was in a rural area. Nearly 2,000 patients per year from many parts of Germany were treated in the clinic, which had 200 beds. Of the patients treated, 15% were university graduates. At the beginning of the treatment programme (average duration of seven weeks), a pre-history based on depth psychology was conducted followed by a medical examination. Based on the results from these evaluations, a psychodynamic treatment was developed and performed by a team of physicians, psychologists, kinesiotherapists, gestalt therapists and nurses. They conducted the treatment mainly in the form of milieu therapy [17]. The stationary psychotherapeutic approach was holistic and included the concept of combining all areas of the clinic as the therapy location. According to the background of a psychoanalytical conception of man and disease, it included not only the psychic but also the somatic and social problems of patients. The centre of the treatment programme was group psychotherapy according to the "Göttingen model" [18].

The diagnostically-open inhomogeneous groups also included members of other occupational groups. In addition to the discussion therapy group meeting twice per week, these patients had three sessions of gestalt therapy and concentrative kinesiotherapy [19]. During these sessions, the participants were able to symbolise their conflicts and problems.

Once per week, the patients came together in a "burnout group". This group discussed concrete everyday problems or the organisation of work to be performed at home with colleagues, school management, pupils and pupils' parents. The members defined the topics to be dealt with. The programme of stress management for teachers elaborated on by Kretschmann in 2001 [20] formed the basis of behaviour therapy in a group setting. Each session was 100 minutes in duration. In addition, two one-on-one depth psychology conversations lasting 50 minutes took place. The topics of these discussions were conflicts, interpretation and work on behaviour and reactions shown during group psychotherapy. The aim was to demonstrate based on several examples how the burnout problem occurs in specific cases and in different situations. Another objective of the individual therapy was to critically analyse the behaviour and activities at work to find a solution.

The main emphasis of this connection was to obtain information about techniques of work and time management as well as the analysis of subjective feelings justifying teachers' behaviour at work, with the objective of changing their attitudes. This procedure was directed towards the patients' resources and to their sound personal characteristics thus therapeutically improving their competence [21]. The initiation of further ambulant therapy and/or the discussion of possibilities of supervision to perform a professional self-reflection at home was another element of the stationary treatment.

The questionnaire included 8 pages with almost 60 questions of varying levels of complexity, covering demographic data, questions about working hours, work organisation, professional history, the duration of incapacity due to illness in the last quarter and the Maslach burnout inventory (MBI) [15,16] in its German translation [22]. The MBI consists of 22 statements about feelings and attitudes that assess the three aspects of burnout: emotional exhaustion (EE, 9 items), depersonalisation (DP, 5 items) and personal accomplishment (PA, 8 items). Each item is measured on a seven-point Likert scale from 0 (never) to 6 (every day). Examples include the following: EE "I feel emotionally drained from my work", DP "I feel I treat some pupils as if they were impersonal objects", PA "I have accomplished many worthwhile things in this job". High degrees of burnout were assumed for EE > 26, DP > 13, and PA < 34 [16]. Questions about the weekly working time (including working hours at home) were for the last working week and were evaluated for comparable conditions (full-time work, uninterrupted by holidays or illness in the last week).

Missing data of the MBI (1.4% of all items; incomplete data sets in 7.5% of first and 9.3% of follow-up surveys) were replaced by calculated personal mean values of the corresponding MBI factor [23] if only one value of the corresponding factor was missing. The answers were evaluated statistically (t-tests with paired random samples to compare the results of survey periods, t-tests with unpaired random samples for group comparisons as well as corresponding Chi square tests to check frequency differences) using the programme Statistica 7 (Statsoft Inc., Tulsa, Oklahoma, USA). Tabular presentations were performed according to gender and school type (high school, other school types). No funding for these studies was obtained.

## Results

**Figure 1:**
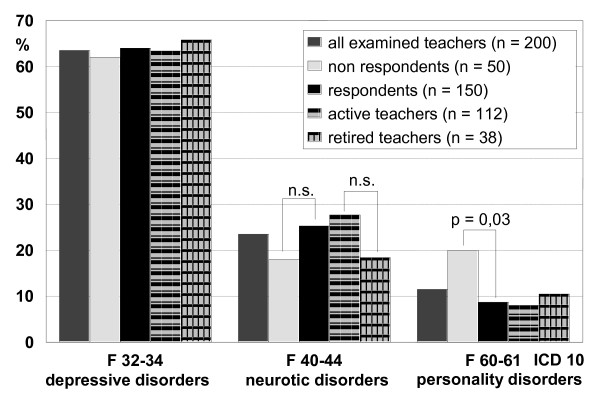
**Rates of main diagnoses in the examined groups of teachers**.

The main diagnoses of the 200 stationary treated teachers were depressive disorders (F 32 - 34; 63.5%), neurotic disorders (F 40 - 44; 23.5%) and personality disorders (F 60 - 61; 11.5%) (Figure 1). The remaining 1.5% (n = 3) had a.o. somatoform disorders. Gender differences existed in the frequency of personality disorders (males 18.2%. females 8.2%; p < .05). The teachers who did not return the follow-up questionnaire showed no significant differences from those who participated twice, except for men with the diagnosis of personality disorders (20% vs. 9%, not returning vs. returning questionnaire).

The percentage of teachers with burnout risk (EE > 26) was in the group tested first between 72.3% (neurotic disorders) and 82.6% (personality disorders, p > .05; depressive disorders 80.3%, total group 78.5%). There was neither an age difference at the first examination nor a distinction of MBI results between responders and non-responders (p > .05).

**Figure 2:**
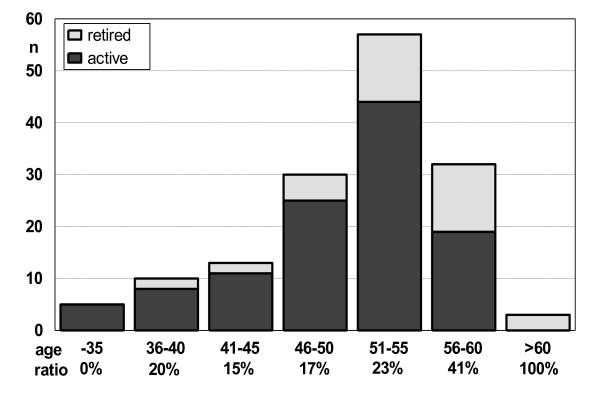
**Age classification of retired and active teachers on the follow-up two years after the therapy, with ratio of retired teachers**.

Out of 150 teachers, who had participated in the follow-up survey 112 (74.7%, males 76.1%, females 74.0%) were still active or had resumed teaching. The percentage of retired or no longer teaching participants increased with age (Figure 2).

**Figure 3:**
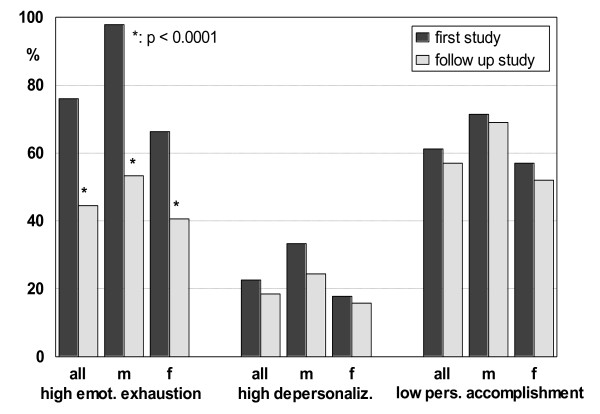
**Percentages of the MBI-subscales in all members of the follow-up study in comparison to their scores during the first study**. m: males. f: females.

The weekly working hours of teachers who had resumed work decreased slightly from 38.1 to 35.5 hours (non-significant). The percentage of those who were not ill in the last quarter increased from 29.5 to 51.8% (p < .001), the number of days off due to illness (all employees) in the last quarter decreased to less than one-third. There was also an essential improvement of MBI scores of EE. Table 1 shows the outcome according to gender and school type. High school teachers showed a statistically significant higher score of emotional exhaustion compared to teachers of other levels (p < .05). The difference disappeared after treatment (p = .599). At the first survey, males had higher EE scores (p < .0001) and DP scores (p < .05) than females and lower PA scores (p < .05); however, males only had higher EE scores (p < .05) at the follow-up survey. In the follow-up survey, female and male participants demonstrated improvements in the subscale values of high EE, high DP, and low PA (Figure 3).

**Table 1:** Comparison between the data obtained during the first study and the follow-up study*; mean values ($\bar{\text{x}}$), standard deviations (s) of age (years) and MBI scores, t-test results (p)

		first study		Follow-up study		
	n	$\bar{\text{x}}$	s	$\bar{\text{x}}$	s	**p**_**2**_
**Disability last quarter [days]**						
all	105	27.5	35.6	8.6	19.1	< .0001
males	34	29.8	39.2	10.0	21.9	.005
females	71	26.4	34.0	7.9	17.8	< .0001
p_1_		.653		.599		
high school	18	34.3	41.8	10.7	22.0	.045
other school types	87	26.1	34.3	8.1	18.6	< .0001
p_1_		.379		.603		

**Emotional Exhaustion [score]**						
all	109	32.0	11.8	25.1	12.6	< .0001
males	34	38.8	8.6	29.5	13.3	< .0001
females	75	28.9	11.8	23.1	11.9	< .0001
p_1_		< .0001		.013		
high school	19	37.7	10.2	26.5	12.5	< .0001
other school types	90	30.8	11.8	24.8	12.7	< .0001
p_1_		.019		.594		

**Depersonalisation [score]**						
all	109	7.9	7.1	7.0	5.9	.085
males	34	10.2	8.0	8.6	6.0	.075
females	75	6.8	6.4	6.2	5.7	.367
p_1_		.021		.052		
high school	19	10.6	7.8	8.9	5.9	.121
other school types	90	7.3	6.8	6.6	5.8	.214
p_1_		.065		.111		

**Personal Accomplishment [score]**						
all	105	27.9	9.0	29.3	8.5	.046
males	31	24.9	9.3	27.9	9.4	.027
females	74	29.2	8.7	29.8	8.2	.409
p_1_		.026		.295		
high school	19	25.7	6.9	26.2	7.6	.622
other school types	86	28.5	9.4	29.9	8.6	.053
p_1_		.229		.086		

*The table contains only individuals who returned to work after therapy

p_1_: Results of t-tests with unconnected random samples in the above groups.

p_2_: Results of t-tests with connected random samples obtained in the same participants during the first study and the follow-up study.

## Discussion

Contrary to other occupational groups, it is easier for German teachers to undergo a qualified treatment for mental exhaustion and health care measures organised under private law but paid by the state due to their status as civil servants. Considering the aspect of recruitment, teachers, a mostly homogeneous group, were best suited to participate in a therapeutic intervention study because they are frequently affected by burnout [5,24,25].

We examined a total of 200 teachers in a period of six years and recorded a complete set of data for this group via medical examination and questionnaire. None of the teachers admitted to the clinic refused to take part. The mainly psychotherapeutic treatment was related to specific occupational problems. Overall, 150 teachers (75%) participated in the follow-up katamnestic survey after approximately two years.

The most important study results are the high quota of teachers who resumed their activities (75%), the essential reduction of days off work due to incapacity to less than one-third and a significant decrease of the percentage of patients endangered by burnout from 76.0% to less than one half (44.5%) measured by the MBI subscale for emotional exhaustion. As international studies [26,27] show, this EE score has been shown to be best suited for the description of burnout risks. Contrary to former studies on teachers performed by us [14], males showed significantly higher scores of emotional exhaustion than females. In our opinion and according to the literature, this finding is the result of a reduced willingness to be treated by psychotherapy or depth psychology. Peterson et al. [28] reported in 2008 that male employees endangered by burnout were rarely prepared to participate in a burnout intervention programme. This attitude is the reason why men tend to decide to undergo treatment much later than women, and probably only when faced by more severe symptoms. With regard to diseases diagnosed according to ICD 10, gender-specific differences were also observed: males were twice as likely to have personality disorders as females. Moreover, these patients returned the questionnaire less frequently than women or employees with main diagnoses of depressive disorders or neurotic disorders. Significant differences between high school teachers, which showed higher risk of burnout, and teachers of other levels could only be found before treatment. After the therapy the differences were not significant anymore. One reason could be the success of the treatment.

The present study is limited by several reasons. First, the number of participating high school teachers compared to those from other school types is relatively narrow. Second, the lack of a control group restricts the results. For all participants a stationary clinical treatment was indicated. Thus, due to ethical considerations the acute symptomatic of the treated teachers would have complicated the realization of a control group setting. We lack information on a comparable extensive intervention study which could present an appropriate stationary control group. Those studies comparing results of treatment and control groups were mostly obtained with ambulant patients, internet-based [29], or by investigations at work [30-32].

## Conclusion

According to our knowledge, our study is the most extensive inpatient intervention study on the burnout of a defined occupational group, i.e., teachers.

The proven and long-term success of the performed intervention could have greater effects if people, especially males, undergo treatment more frequently. Our results are based on selectively high proposition of teachers of advanced age. Thus it is possible that the long term effect of the intervention, particularly on retirement age, is greater when the intervention is started earlier. This aspect should be dealt with in future studies. Regular burnout tests could help to identify risk cases among teachers at an early stage and to offer a therapeutic intervention.

## Competing interests

The authors declare that they have no competing interests.

## Authors' contributions

RW, PB, BP, UM and XB were involved in the conception and design of the study, interpretation of data and critical revisions of the manuscript. RW drafted the manuscript. Vocal informed consent was obtained from the participants of this study. All authors have read and approved the final manuscript.
